# Can We Use Peer-Assisted Learning to Teach Basic Surgical Skills?

**DOI:** 10.21315/mjms2020.27.5.10

**Published:** 2020-10-27

**Authors:** Mang Ning Ong, Kar Min Lew, Yih Jeng Cheong, Evelyn Wan Xuan Ting, Bakri Bohari, Tang Yita, Kandasami Palayan

**Affiliations:** International Medical University, Seremban, Negeri Sembilan, Malaysia

**Keywords:** clinical competence, peer group, minor surgical procedures, suturing suture techniques, education, medical, undergraduate

## Abstract

**Background:**

It is reported that medical students do not receive adequate opportunities to learn surgical skill and are at risk of being unable to perform simple surgical procedures safely. The usefulness of peer-assisted learning (PAL) as a tool to assist in delivering surgical skills training is worth exploring.

**Methods:**

This is a randomised single blinded controlled trial. Fourth-year students from the university’s Surgical Society were asked to volunteer as peer tutors and those in 3rd-year were asked to undertake surgical skills training. A cohort of 35 students were selected and randomised to receive basic surgical skills training conducted either by faculty members or peers. The students’ performance of basic suturing skills was assessed using a checklist, through directly observed procedural skills (DOPS) technique. The assessment was conducted by faculty blinded to the training. Students’ perception to surgical skills training was assessed using a questionnaire survey.

**Results:**

The suturing and knotting skills of students learned from their peers was comparable to that acquired from faculty. The students’ perceived that their peers could conduct surgical skills training similar to their faculty.

**Conclusion:**

PAL approach for basic surgical skills training is as effective as faculty-led training. PAL has the potential to optimise the delivery of surgical skills training in undergraduate medical education.

## Introduction

Freshly qualified medical graduates are expected to be competent in practical skills such as suturing of simple wounds. Unfortunately, surveys have noted that they lack the ability to perform these simple practical procedures (1, 2). There is increasing evidence that suggest medical students do not receive enough training in basic surgical skills. In a national review of surgical skills training in the United Kingdom, it was revealed that medical schools provide minimal basic surgical skills training and that the newly qualified doctors are at risk of being unable to safely perform simple surgical procedures (3, 4). There is also evidence from Europe and Australia that indicate medical schools do not provide sufficient surgical skills learning opportunities for undergraduates (5). In our own review in Malaysia, we noted that medical graduates failed to demonstrate the desired level of competencies in practical skills (6). The teaching of basic surgical skills is demanding as it utilises a considerable amount of faculty time and resources of the department. The logistics of delivering basic surgical skills repetitively and regularly to different cohort of students is particularly challenging. Medical students often find the training opportunities hampered and compensate for inadequacies by attending informal surgical skills workshops organised by Surgical Societies and other professional bodies (7).

Peer-assisted learning (PAL) is an established teaching and learning method in medical education. Worldwide, medical schools use PAL, whereby students in senior years teach other students in more junior years. Globally, interest in PAL during undergraduate medical programmes has gained increasing attention in recent years. It has been suggested that PAL can potentially achieve equivalent learning outcomes compared to conventional teaching (8). Using PAL as an adjunct to optimise surgical skills learning in undergraduate medical education may be a useful consideration. Unfortunately, there is little information in the literature that explores PAL to supplement undergraduate surgical skills training. Incidentally, at International Medical University (IMU) in Malaysia, students in the Surgical Society conduct informal surgical skills training for their peers. This study aims to determine if PAL approach to basic surgical skills training can be as effective as skills training led by surgical faculty. Also, our additional aim is to determine students’ perception towards PAL approach to surgical skills training.

## Methods

### Settings

This study was conducted at the clinical campus of IMU where basic surgical skills’ training is an integral part of the surgical curriculum. Surgical skills such as handling of surgical instruments, knotting and suturing skills are formally introduced from year 3 onwards. The surgical skills learning is structured and conducted in the skills learning centre using bench models. In addition to the formal skills training, the Surgical Society of IMU (IMUSS) regularly conducts PAL skills training programme for their peers on an informal basis. Peer tutors generally consist of 4th-year students who are members of the IMUSS.

### Tutors

Fourth-year students who were members of the IMUSS were eligible for the task of peer tutors in this study. To minimise bias in the intervention arm, students were asked to volunteer to be peer tutors, rather than being handpicked. Members of the IMUSS were preferred as peer tutors because they generally show interest in surgery and enjoy peer tutoring. The peer tutors received instructions on the goals and objectives of surgical skills training sessions. The peer tutors did not receive any additional training on how to conduct the teaching of basic surgical skills to their juniors. Members of faculty who were regularly roistered in the timetable to teach surgical skills were requested to teach the students in control arm of the study.

### Trainees and Skills Training

Third-year medical students who had no previous exposure to surgical skills learning were asked to volunteer to participate in the study. Thirty-five students were recruited and randomised to receive basic surgical skills training either by surgical faculty members (Group A) or peer tutors (Group B). Surgical skills training for both the groups were standardised and consisted of two sessions, each of 2 h duration. In the first session, students learned about wound care, suture materials, knotting techniques and the handling of surgical instruments. In the second session, the students learned and practiced wound closure using interrupted and continuous technique by chicken skin bench model. Each training session consisted of didactic teaching, live demonstration and practice of surgical skills by students. Skills training was conducted by 2–3 tutors, and trainees were closely supervised and provided appropriate feedbacks.

### Assessment of Surgical Skills

Students’ performance on basic surgical skills was assessed using Direct Observation of Procedural Skills (DOPS) technique. Two skills stations were used. In skills station 1, students were required to demonstrate their proficiency in performing an interrupted wound closure technique and knot tying using an instrument. In skill station 2, students’ proficiency in the closing of a 3 cm wound using continuous wound closure technique and knot tying with hands were assessed. A checklist vetted by the department’s Vetting Committee was used to assess the practical skills. The domains assessed the skills stations, included handling of surgical instruments, suturing technique, tissue handling, knotting and the overall performance. The assessors of the procedural skills were blinded and were ignorant of the training the students received.

### Questionnaire

Following the workshop, students completed a questionnaire to assess the students’ perception of the tutors in imparting the surgical skills. The domains assessed in the questionnaire included: i) quality of tutors; ii) teaching methods; iii) training environment; and iv) trainees’ confidence of the training. The 5-point Likert scale (strongly disagree to strongly agree) was used to assess students’ perception. It provided space for text comments on skills training.

### Statistical Analysis

An independent samples *t*-test was used to analyse the theoretical test and DOPS score. Paired *t*-test was used to compare between instrument tie and hand tie and between interrupted and continuous wound closure. Mann-Whitney U test was used to analyse the five-point Likert score used for questionnaires. All statistical analyses were conducted using IBM SPSS Statistics version 20.

## Results

### Assessment of Suturing Skills

In this study, a total of 35 third-year medical students were randomised to receive either faculty-led (*n* = 18) or peer-led (*n* = 17) skills training. All the students completed their training and underwent assessment of their proficiency in closing surgical wounds and tying of square knots. Table 1 shows the mean scores of student’s proficiencies in closing a surgical wound using interrupted suturing technique and tying square knots using an instrument. Table 1 also compares the above-mentioned details between the faculty-led group and the peer-led group. The mean score of suturing skill of the peer-led group was slightly higher than that of the faculty-led counterparts (7.71 ± 1.68 versus 7.08 ± 1.75), but the difference was not statically significant. In the skill relating to the handling of tissues, the peer-led group had higher mean scores (1.85 ± 0.79) as compared to the faculty (1.28 ± 0.65) (*P* < 0.05). However, no statistical difference was seen in the mean scores between the two groups in skills related to handling of instruments and the tying of a surgical knot with an instrument. The overall performance in interrupted wound closure technique and knot tying with an instrument of the peer-led group was comparable to that led by faculty (15.91 ± 3.12 versus 14.75 ± 3.58). Though the faculty-led group had a slightly higher mean score, this was not statistically significant.

Table 2 compares the mean scores of students’ proficiency in closing a surgical wound using continuous suturing technique and tying square knots using hands of the faculty-led group with that of the peer. In contrast to Table 1, students in the faculty-led group performed better than the peer counterparts in the suturing technique (10.42 ± 2.56 versus 9.79 ± 3.41). However, this was not statistically significant. The overall performance in continuous suturing technique and tying square knots using hands of the peer-led group was comparable to that led by faculty (13.85 ± 4.90 versus 13.94 ± 3.51).

In Table 3, although the scores for knotting skills in peer-led group was comparable to that of the faculty, the students’ scores for knot tying using instruments was significantly higher than scores for knot tying using hands in both the groups. Students appeared to be more proficient in tying knots using instruments rather than tying knots with hands, irrespective of the instructor.

In Table 4, although the scores for continuous and interrupted wound closure technique in peer-led group was comparable to that of the faculty counterparts, there was a significant difference in the scores for continuous wound closure technique knot when compared to interrupted wound closure in both the groups. The students appeared to be more proficient in continuous wound closure technique as compared interrupted wound closure, irrespective of the instructor.

The response rate for the 10-item questionnaire was 100%. Table 5 compares the mean scores of students’ perception on peer-led surgical skills training to faculty-led training using the 5-point Likert scale. The students’ overall perception of surgical skills training was positive for both groups. There was no statistical difference between the scores from the two groups of students. In the free text column, the students noted that the training environment of the peer-led group was more conducive and peers were very helpful.

## Discussion

Training in basic surgical skills is recognised as an essential component of the undergraduate medical school curriculum. Unfortunately, evidence suggest that medical students do not receive adequate surgical skills learning opportunities and are at risk of being unable to perform simple surgical procedures safely (3). Traditionally, medical students learned their surgical skills by watching seniors in the operation theatre or other clinical settings, and then copied and practiced them on real patients (10). In consideration of patient safety and ethical values, apprenticeship model of learning practical skills on real patients is no longer acceptable. In recent years, medical schools have embraced laboratory-based surgical skills training (11). Surgical skill training is standardised and delivered in a structured and objective manner to small groups of students. Although the new paradigms have produced major improvement in the quality of basic surgical-skill education, medical schools struggle to provide adequate opportunities for training. The teaching of basic surgical skills is demanding and requires a significant obligation on the time-constrained faculty. There is a need to design new approaches and optimise basic surgical skills training opportunities for undergraduate medical students. Medical schools worldwide use PAL effectively in their teaching and learning activities (8, 9). At our institution, students in the Surgical Society regularly conduct surgical skills workshops for their peers as an adjunct for their regular classes. This experience has allowed us to explore the creation of new educational environments to strengthen surgical skills learning opportunities. This study has shown that students who learned wound closure techniques from their peers and in the traditional way from lecturers did well. This finding indicates that PAL can be used as an effective tool in the delivery of basic surgical skills training. In this study, we have also examined the perceptions of students to PAL in surgical skills training in comparison to established faculty training. Interestingly, the findings indicate that students embraced PAL as an effective training strategy. They found PAL training sessions are more relaxed, interactive and enjoyable. Our experience indicates that PAL surgical skills training sessions has a potential to be incorporated in our undergraduate medical curriculum. The uniqueness of this study is not only the delivery of surgical training but that we were also able to determine if this method was effective in imparting the desired level of competencies.

We used DOPS, a structured assessment tool, to evaluate the students’ competency in the handling of surgical instruments, tissues, suturing technique and knot tying skill. DOPS is an effective and efficient evaluation method used for assessing competence in practical procedures that trainees undertake (12). In this study, the performance of the students in the skills stations demonstrates how well they learnt the skills during the training. If they have been trained well and acquired the practical skills, their performance will be reflected by a higher mean score.

Our evaluation showed that the overall mean score of the performance of students trained by their peers was comparable to those who were coached by faculty members. The acquisition of basic surgical skills after PAL appears to be comparable to the training supervised by surgical faculty surgeon.

The study has demonstrated that the PAL surgical skills training is as effective as the training conducted by faculty.

Interestingly, this study highlights the significance of establishing a formal assessment system, a key component of surgical skills learning. The study demonstrates that despite the formal training, either by faculty or by their peers, the students were not proficient in certain specific skills. We noted that students, irrespective of the instructor, were more proficient in tying knots with instruments as compared to tying knots with hands. This was reflected in the significantly higher score obtained by students for instrument tie as compared to those done by hand. The students also demonstrated that they were more proficient in performing wound closures using continuous suturing rather than interrupted suturing technique. This was evidenced by significantly higher scores in performing wound closure using continuous suturing (10.11 ± 2.97) as compared to interrupt suturing technique (7.39 ± 1.72). The assessment exposed the deficiencies within training sessions; students may benefit from more focused attention in these specific skills in future training sessions. For skills training to be effective, irrespective of whether it is peer-led, or faculty-led, the trainees must receive appropriate supervision, guidance and enough time for practice. Moreover, it is important to recognise that inadequate supervision in training has the potential for trainees to develop bad habits that are more difficult to unlearn in later years. We propose that medical schools should closely monitor the delivery of surgical skills training and establish systems to formally assess the effectiveness of the training programme.

Lack of undergraduate surgical skills training contributes to medical graduates’ unpreparedness to perform basic practical tasks in clinical practice. Medical schools must acknowledge that medical graduates must have appropriate competency in performing basic surgical procedures such as suturing. Institutions that lack the capacity and capability to deliver surgical skills training must explore new approaches to overcome the shortfalls. Our study is limited by its small sample size; nevertheless, the encouraging results justify a larger prospective investigation. The effectiveness of peer-led training, as a new tool to improve surgical skills training is worth pursuing. PAL has the potential to be a reliable approach in ensuring effective undergraduate surgical skills training. Students find PAL environment to be less intimidating and daunting. There are also encouraging evidence to suggest that PAL improves students’ confidence and ability in performing basic surgical skills and contributes to strengthening students’ desire to pursue a surgical career. Student tutors also benefit from teaching as they are able improve their individual knowledge and skills and develop leadership qualities. Medical students should be given the opportunity, resources and encouragement to contribute for training of their colleagues.

## Conclusion

PAL is an effective educational tool to improve medical student proficiency in performing basic surgical procedures. The surgical fraternity must recognise the significance of PAL in surgical skills training and explore its potential for it to be incorporated into the surgical curriculum.

## Figures and Tables

**Table 1:** Students’ proficiency in wound closure using interrupted suturing technique and knot tying with an instrument

Skills	Maximum score	Faculty-led group (*n* = 18)	Peer-led group (*n* = 17)	*P*-value*
Instrument handling skill	3.0	1.94 ± 0.64	2.00 ± 0.87	0.83
Suturing skill	14.0	7.08 ± 1.75	7.71 ± 1.68	0.29
Tissue handling skill	2.0	1.28 ± 0.65	1.85 ± 0.79	0.02
Knotting skill	3.0	2.39 ± 0.98	2.41 ± 0.71	0.94
Overall performance	3.0	2.06 ± 0.80	1.94 ± 0.66	0.65

Total	25.0	14.75 ± 3.58	15.91 ± 3.12	0.31

Notes: A: maximum score available for each skill domain; B: value given as mean ± standard deviation;

* *P* < 0.05 considered statistically significant

**Table 2:** Students’ proficiency in wound closure using continuous suturing technique and hands to tie knots

Skills	Maximum score	Faculty-led group (*n* = 18)	Peer-led group (*n* = 17)	*P*-value*
Instrument handling skill	3.0	1.19 ± 0.35	1.09 ± 0.92	0.66
Suturing skill	14.0	10.42 ± 2.56	9.79 ± 3.41	0.54
Tissue handling skill	2.0	0.78 ± 0.62	1.06 ± 0.61	0.19
Knotting skill	3.0	0.28 ± 0.67	0.47 ± 1.18	0.55
Overall performance	3.0	1.28 ± 0.57	1.44 ± 0.50	0.66

Total	25.0	13.94 ± 3.51	13.85 ± 4.90	0.95

Notes: Value given as mean ± standard deviation;

* *P* < 0.05 considered statistically significant

**Table 3:** Comparison of square knot tying (instrument versus hand)

Groups	Knotting skill		*P*-value**
	Hand tie	Instrument tie	
Faculty-led (*n* = 18)	0.28 ± 0.67	2.39 ± 0.98	< 0.01
Peer-led (*n* = 17)	0.47 ± 1.18	2.41 ± 0.71	< 0.01
*P*-value*	0.55	0.94	

Notes: Value given as mean ± standard deviation;

* *P* < 0.05 considered statistically significant, obtained using independent samples *t*-test;

** *P* < 0.05 considered statistically significant, obtained using paired samples *t*-test

**Table 4:** Comparison of interrupted and continuous wound suture technique

Groups	Suturing skill		*P*-value**
	Continuous suture technique	Interrupted suture technique	
Faculty-led group (*n* = 18)	10.42 ± 2.56	7.08 ± 1.75	< 0.01
Peer-led group (*n* = 17)	9.79 ± 3.41	7.71 ± 1.68	< 0.01
*P*-value*	0.54	0.29	

Notes: Value given as mean ± standard deviation;

* *P* < 0.05 considered statistically significant, obtained using independent samples *t*-test;

** *P* < 0.05 considered statistically significant, obtained using paired samples *t*-test

**Table 5:** Trainees’ perception towards basic surgical skills

Domains	Groups		*P*-value*
	Faculty-led group (*n* = 18)	Peer-led group (*n* = 17)	
Ability to impart objective clearly	4.61 ± 0.61	4.29 ± 0.59	0.09
Quality of tutors	4.33 ± 0.77	3.94 ± 0.90	0.15
Teaching methods	4.89 ± 0.32	4.71 ± 0.47	0.18
Training environment	3.89 ± 0.83	3.94 ± 0.83	0.83
Trainees confidence of the training	4.28 ± 0.67	3.94 ± 0.90	0.32
Total	22.00 ± 2.66	20.82 ± 2.65	0.90

Notes: The student perceptions were ranked on a 0 to 25 Likert scale, with 5 representing strongly agree on a Likert item; scores were expressed as mean ± SD;

* *P* < 0.05 considered statistically significant; taken from Mann-Whitney U test except total scores taken from independent sample *t*-test
